# Oncogenic MicroRNAs: miR-155, miR-19a, miR-181b, and miR-24 enable monitoring of early breast cancer in serum

**DOI:** 10.1186/1471-2407-14-448

**Published:** 2014-06-18

**Authors:** Marek Sochor, Petra Basova, Michal Pesta, Nina Dusilkova, Jiri Bartos, Pavel Burda, Vit Pospisil, Tomas Stopka

**Affiliations:** 1 Comprehensive Cancer Centre, Regional Hospital Liberec, Liberec, Czech Republic; 2 1st Faculty of Medicine, Institute of Pathological Physiology, Charles University in Prague, Prague, Czech Republic; 3 Faculty of Mathematics and Physics, Charles University in Prague, Prague, Czech Republic

**Keywords:** Breast cancer, microRNA, miR-155, miR-19a, miR-181b, miR-24, Let-7a

## Abstract

**Background:**

MicroRNAs (miRs) represent a distinct class of posttranscriptional modulators of gene expression with remarkable stability in sera. Several miRs are oncogenic (oncomiRs) and are deregulated in the pathogenesis of breast cancer and function to inhibit tumor suppressors. Routine blood monitoring of these circulating tumor-derived products could be of significant benefit to the diagnosis and relapse detection of early-stage breast cancer (EBC) patients.

**Methods:**

Aim of this project was to determine expression of miR-155, miR-19a, miR-181b, miR-24, relative to let-7a in sera of 63 patients with EBC and 21 healthy controls. Longitudinal multivariate data analysis was performed to stochastically model the serum levels of each of the oncomiRs during disease phases: from diagnosis, after surgery, and following chemo/radiotherapy. Moreover, this analysis was utilized to evaluate oncomiR levels in EBC patients subgrouped using current clinical prognostic factors including HER2, Ki-67, and grade III.

**Results:**

EBC patients significantly over-express the oncomiRs at the time of diagnosis. Following surgical resection the serum levels of miR-155, miR-181b, and miR-24 significantly decreased (*p =* 1.89e-05, 5.41e-06, and 0.00638, respectively) whereas the miR-19a decreased significantly after the therapy (*p =* 0.00869). Furthermore, in case of high-risk patients serum levels of miR-155, miR-19a, miR-181b, and miR-24 are significantly more abundant in comparison to low-risk group (p = 0.026, 0.02567, 0.0250, and 0.00990) and show a decreasing trend upon therapy.

**Conclusions:**

OncomiRs are significantly more abundant in the sera of EBC patients compared to controls at diagnosis. Differences in oncomiR levels reflecting EBC risk were also observed. Testing the oncomiRs may be useful for diagnostic purpose and possibly also for relapse detection in follow-up studies of EBC.

## Background

Left untreated, early-stage breast cancer (EBC) can progress into a deadly disseminated cancer and thus early detection is of utmost importance. In addition to BRCA mutations that occur within a relatively small patient cohort there exists other pathology risk factors such as hormonal receptors, HER2, Ki-67, and grade III that aide in determining the potential risk of disease progression or relapse. MicroRNAs (miRs) are additional factors that are related to tumor growth and are detectable in a patient specimen [1]. MicroRNAs are 18–24 nucleotide-long non-coding RNAs each of them able to inhibit hundreds of mRNAs. MicroRNAs are produced as genes and function to inhibit their target mRNAs based on complementarity, thus take part in normal cellular processes as well as BC oncogenesis [2]. MicroRNAs are exported outside the cells and due to their stability are readily detectable in peripheral blood samples. This feature can be very important for monitoring of miRs secreted from the expanding EBC tissue.

Work by others involving 76 BC samples and 10 control tissues and utilizing microarray technique followed by Northern blot validations demonstrated that miRs are deregulated in BC with a defined ‘signature’ often observed; decreased: miR-10b, miR-125b, miR-145, increased: miR-21, and miR-155 [3]. For example, expression of miR-155 in BC (normalized median = 1.75, range 0.95-11.45) and in control tissue (1.37, 1.11-1.88) was found significantly different according to ANOVA [3]. Moreover, additional oncomiRs such as miR-17-92 cluster is also deregulated in BC and contributes to transformed BC characteristics [4] possibly through significant changes in target gene expression [5]. Several oncomiRs are potentially involved in aggressiveness including let-7b, miR-30c, miR-148a, miR-181a, miR-181a*, and miR-181b based on next generation sequencing study of several cases of highly invasive micropapillary carcinoma [6].

Notably, several miRs including miR-24 and miR-181b displayed co-upregulation in sera and tumor tissue of BC patients (N = 13) compared to controls (N = 10) using sequencing approach [7]. While several miRs are repeatedly deregulated others are stable and show very little variability in sera such as: let-7a, miR-16, miR-93, miR-103, miR-192, and miR-451 in a study of gastric cancer patients [8] or: let-7a and miR-195 in BC patient plasma as compared to normal controls [9]. Let-7a levels are stable likely because it is widely expressed in somatic cells, while less expressed in rare stem cells and undifferentiated precursors [10]. In agreement, let-7a was found slightly downregulated in several malignancies including breast cancer samples [11] and upon BC tumorigenesis [12,13].

Based on above-mentioned data the miR-155, miR-19a, miR-181b, and miR-24 represent very good candidates to monitor tumor growth in BC patients as serum molecular biomarkers (albeit there are possibly other oncomiRs involved in BC pathogenesis, aggressiveness, or tumor growth). A direct role of these oncomiRs in BC pathogenesis is supported by studies on their target genes respectively. For example, the oncomiR miR-155 is overexpressed in BC and functions to downregulate *Suppressor of cytokine signaling-1* (SOCS1) leading to persistent activation of the *Signal Transducer and Activator of Transcription-3* (STAT3) [14]. Indeed, upregulation of miR-155 upon diagnosis of BC and a decrease of its levels upon therapy was previously observed based on data using the spike-in control [15]. Interestingly, the decline of serum miR-155 levels following therapy was observed much earlier than that of other tumor-biomarkers [15]. Next, miR-19a, a member of the miR-17-92 cluster, affects BC pathogenesis by multiple mechanisms including inhibition of tumor suppressor PTEN [16]. Involvement of miR-181b is suggested in aggressive BC due to its role in DNA damage response by downregulating ATM and signal efficiency of PARP1 inhibitors in the treatment of triple-negative BC [17]. Recently, expression of miR-19a and miR-181b has been interlinked by a study showing that miR-19a-mediated inhibition of SOCS1 activates miR-181b expression (through STAT3) [18]. In BC pathogenesis, role of TGFβ-mediated epithelial-to-mesenchymal transition has been repeatedly suggested with a focus on miR-24 role in this process [19]. Taken together, microRNAs selected for this study are key molecules involved in tumor growth and aggressiveness of BC.

We herein studied expression of miR-155, miR-19a, miR-181b, and miR-24 in sera of 63 EBC patients and evaluated statistically significance of their expression throughout the therapy and in relation to clinical risk stratification. Our work suggests that the serum-oncomiR expression associates with EBC at diagnosis especially in high-risk patients and can be potentially useful for disease monitoring.

## Patients, materials, and methods

### Patient inclusion and sample collection

Patient sera, collected in years 2010–2013 from EBC (N = 63, median age 58) was obtained following written informed consent based on Helsinki declaration and approved by *Ethics Committee of the Regional Hospital in Liberec, Czech Republic* (under #EK/83/2010). Written informed consent for publication of patients clinical details was obtained. EBC is defined as a BC that has not spread beyond the breast or the axillary lymph nodes (this includes ductal carcinoma in situ and invasive BC stages I, IIA, IIB and IIIA). Timing of sera collection in each patient was: one day prior operation (time point I), 14–28 days after operation before any non-surgical treatment (time point II) and 14–28 days after first treatment modality: either chemotherapy or radiotherapy (time point III). The surgery involved in all patients the tumor removal together with the successful removal of surrounding non-tumorous tissue. Median follow-up was 27 months (ranges 9.5-36.3) (see Additional file 1). Upon clinical relapse the patients (N = 3) donated another serum sample (time point IV). Diagnosis and therapy decisions were made using standard criteria and were not influenced by any results presented in this manuscript. Blood samples were collected into 10 cc tubes with polymer gel and clot activator (BD Vacutainer SST™ Tubes), allowed to clot in room temperature for 30–60 minutes, spun at 3000 rpm for 10 minutes, and aliquoted and placed into −80°C freezer.

Clinical parameters of EBC patients include age, menopausal status, personal cancer history, histological diagnosis, clinical and surgical stage, tumor type, tumor grade, hormonal receptor status, Ki-67, and HER2 expression (see Additional file 1). High-risk group patients presented with one or more of the following characteristics: triple-negativity (ER, PgR and HER2 negative), HER2 positivity, grade III, Ki-67 ≥ 20%, and axillary lymph-node positivity. Low-risk patients were negative for these characteristics. Normal serum samples were randomly collected from 21 healthy female-volunteers (age ranges 25–60 years) during the same time period. Control sera were obtained following written informed consent based on Helsinki declaration and approved by *Ethics Committee of the Regional Hospital in Liberec, Czech Republic* (under #EK/83/2010). Written informed consent for publication of healthy controls clinical details was also obtained.

### Sample processing and miRNA extraction and quantitation

RNA was isolated using miRNeasy® Mini Kit (Qiagen) from 200 μL of sera (lysed by 1 ml of QIAzol® Lysis reagent) with several modifications including a) vigorous vortexing after mixing with CHCl_3_, b) centrifugations at 10800 rpm/15 min/at 4°C, c) use of glycogen during ethanol precipitation, d) multiple (3×) washes with 500 μl RPE buffer, and e) elution volume to be 40 μl of DNAse&RNAse-free water. RNA level was too low to be detectable by NanoDrop. Reverse transcription was performed using High Capacity cDNA Reverse Transcription Kit supplemented with miR-specific primers (Life Technologies, USA). Quantitative polymerase chain reaction (PCR, using the ABI 7900HT instrument) was run 40 cycles of 95°C for 15 seconds and 60°C for 1 minute. Relative expression was calculated from CTs of the oncomiR (miR-155, miR-19a, miR-181b, miR-24) relative to let-7a in EBC *vs* healthy sera using 2^-(ΔCT)^ equation. Mean values and the standard deviation of the dCT values (of each sample and average control sample) of reference microRNA let-7a in the patient samples at each collection time (I-III) and in the control samples document that let-7a is stably expressed in the serum samples (mean_I_ = −0.06818 stdeva_I_ = 0.6640; mean_II_ = −0.01103 stdeva_II_ = 0.8948; mean_III_ = 0.01014 stdeva_III_ = 0.8488; mean_CTRL_ = 0.05397 stdeva_CTRL_ = 0.6604).

### Statistical analysis of the serum levels of oncogenic miRs in BC patients over therapy

Serum levels of the oncogenic miRs (miR-155, miR-19a, miR-181b, and miR-24, all relative to let-7a) are recorded for each BC patient at three time points over the therapy: diagnosis (I), after surgical resection (II), and after chemo- and/or radio-therapy (III). Serum levels in case of female controls are measured only once, i.e., at time point I (control diagnosis).

Longitudinal multivariate data analysis is performed to stochastically model the serum levels of each of the miRs. The following random effects model is used for miR-19a and miR-181b

$$\begin{array}{l} E\left[\mathrm{mi}R_{i,t}|b_{i}\right]=1/\{\beta_{I}+\beta_{\mathrm{II}}\left(\mathrm{Patien}t_{i}\;\mathrm{in}\;\mathrm{II}\right)\\ \;+\beta_{\mathrm{III}}\left(\mathrm{Patien}t_{i}\;\mathrm{in}\;\mathrm{III}\right)+\beta_{C}\left(\mathrm{Contro}l_{i}\right)\\ \;+\beta_{L}\left(\mathrm{LowRis}k_{i}\right)+b_{i}\}, \end{array}$$

and for miR-155 and miR-24

$$\begin{array}{l} E\left[\mathrm{mi}R_{i,t}|b_{i}\right]=\exp\{\beta_{I}+\beta_{\mathrm{II}}\left(\mathrm{Patien}t_{i}\;\mathrm{in}\;\mathrm{II}\right)\\ \;+\beta_{\mathrm{III}}\left(\mathrm{Patien}t_{i}\;\mathrm{in}\;\mathrm{III}\right)+\beta_{C}\left(\mathrm{Contro}l_{i}\right)\\ \;+\beta_{L}\left(\mathrm{LowRis}k_{i}\right)+b_{i}\}. \end{array}$$

Here, *E*[*miR*_*i*,*t*_|*b*_*i*_] is the conditional expectation of the miR’s serum level *miR*_*i*,*t*_ for each

BC patient/control identification *i* at time point *t* = *I*, *II*, *III* given random intercept *b*_*i*_, which is specific for each BC patient/control and has a multivariate normal distribution with zero mean. The unconditional distribution of the miR’s serum level is a Gamma distribution.

The mathematical syntax of expression, e.g., (*Patient*_*i*_ *in II*), is that it equals one if and only if the *i*th BC patient/control is in time point II (and, hence, is not a control); zero otherwise.

## Results

### Longitudinal multivariate data analysis model to evaluate oncomiR levels in EBC

We determined the levels of oncomiRs in sera relative to let-7a and obtained a set of values from one day prior operation (time point I), 14–28 days after operation before any non-surgical treatment (time point II) and 14–28 days after first treatment modality: either chemotherapy or radiotherapy (time point III). These values (as shown in Additional file 2: Results 1) show that a large portion of the patients at time points I & II displayed differences when compared to healthy control and time point III samples. In order to statistically evaluate the data, we constructed and used *Longitudinal multivariate data analysis model* (see Methods) for each of the oncomiR. This enabled the analysis of each oncomiR levels within EBC patient sera with respect to time points and risk factors. Additionally, we aimed to define the expected level of each oncomiR at each time point.

The parameters of the longitudinal data model (i.e., estimates of parameters *β*s) are shown in Table 1A-D (miR-155 in A, miR-19a in B, miR-181b in C, and miR-24 in D). *β*_*I*_ represents oncomiR level at time point I, *β*_*II*_ compares oncomiR levels between time points I and II, *β*_*III*_ reflects the difference between time points I and III *β*_*C*_, stands for the difference between time point I and healthy control. Finally, *β*_*L*_ represents the difference between data from high-risk and low-risk EBC patients. Estimated parameter values are used to calculate conditional expectation of the miR’s serum level for each BC patient/control at particular time point (see equations in Methods).

**Table 1:** Summary of the multivariate longitudinal data analysis

**A) miR-155**			
**Parameter**	**Estimate**	**Standard error**	***p*-value**
*β*_*I*_	0.61774	0.06440	< 2e-16
*β*_*II*_	−0.24024	0.05617	1.89e-05
*β*_*III*_	−0.34474	0.05684	1.32e-09
*β*_*C*_	−0.51148	0.11103	4.10e-06
*β*_*L*_	−0.18353	0.08245	0.026
**B) miR-19a**			
**Parameter**	**Estimate**	**Standard error**	***p*-value**
*β*_*I*_	0.60941	0.04843	< 2e-16
*β*_*II*_	0.03224	0.03187	0.31183
*β*_*III*_	0.09981	0.03804	0.00869
*β*_*C*_	0.34506	0.11439	0.00256
*β*_*L*_	0.15711	0.07041	0.02567
**C) miR-181b**			
**Parameter**	**Estimate**	**Standard error**	***p*-value**
*β*_*I*_	0.70370	0.05176	< 2e-16
*β*_*II*_	0.17814	0.03917	5.41e-06
*β*_*III*_	0.17263	0.03884	8.80e-06
*β*_*C*_	0.27792	0.10095	0.0059
*β*_*L*_	0.16588	0.07403	0.0250
**D) miR-24**			
**Parameter**	**Estimate**	**Standard error**	***p*-value**
*β*_*I*_	0.50276	0.06718	7.19e-14
*β*_*II*_	−0.14807	0.05429	0.00638
*β*_*III*_	−0.12527	0.05527	0.02343
*β*_*C*_	−0.34034	0.11512	0.00311
*β*_*L*_	−0.22631	0.08774	0.00990

Presented are parameters to calculate conditional expectation of the miR’s serum level for each BC patient/control at particular time point and their estimate values, their standard errors, and corresponding *p*-values for levels of miR-155 (A), miR-19a (B), miR-181b (C), and miR-24 (D), all relative to let-7a, in EBC sera. Parameters of oncomiR level: *β*_*I*_: time point I, *β*_*II*_: the difference between time points I and II, *β*_*III*_: the difference between time points I and III, *β*_*C*_: the difference between time point I and healthy control, and *β*_*L*_: the difference between high risk and low risk EBC patients.

To exemplify this model; the prediction of miR-19a after therapy for a high-risk BC patient, the predicted serum level of that miR is 1/{0.60941 + 0.09981} ≈ 1.410 (see parameter estimates from Table 1B). On the other hand, the predicted serum level of miR-24 for a low-risk BC patient after surgical resection is exp{0.50276 + (−0.14807) + (−0.22631)} ≈ 1.137 (see Table 1D).

### Levels of oncomiRs decrease after surgery and adjuvant therapy of EBC patients

**Figure 1:**
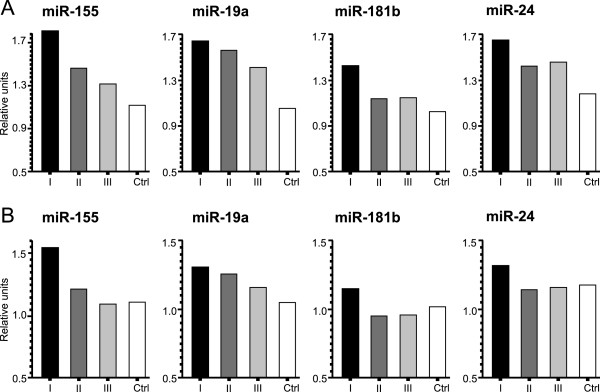
**Conditional expectation of the serum level of miR-155, miR-19a, miR-181b, and miR-24 through the therapy in case of a common BC patient (high-risk patients in upper A panel, low-risk patients in bottom B panel) and its comparison to the control.** The predicted development (mainly decrease) of the serum level for each oncomiR is calculated using the multivariate longitudinal data analysis introduced within Methods and Results sections and also provided in the Table 1.

Using the longitudinal multivariate data analysis we asked whether levels of oncomiRs are different at EBC time point I compared to other time points or controls. Firstly, we determined whether surgical removal of a tumor had any impact on serum oncomiR levels. Between time points I and II the serum levels of miR-155, miR-181b, and miR-24 significantly decreased after the surgical resection (*p*-values < 0.05, i.e., 1.89e-05, 5.41e-06, and 0.00638, respectively) for all (low- and high-risk) EBC patients. *p*-values are shown in the Table 1A-D. Unlike these three oncomiRs (miR-155, miR-181b, and miR-24), serum levels of miR-19a did not change after surgery indicating that either it is more stable or its production does not reflect loss of tumor tissue. However, a significant drop of serum level of each miR (including miR-19a) was noted after the therapy at time point III compared to the time point I at diagnosis (*p*-values < 0.05, i.e., miR-155: 1.32e-09, miR-19a: 0.00869, miR1-81b: 8.80e-06, and miR-24: 0.02343, respectively). It is thus more likely that the absence of any change in miR-19a serum levels upon surgery is due to a stability issue rather than miR-19a production by the non-tumor tissue. Data are comprehensively presented by the Figure 1 and the Table 1.

In summary, the oncomiR levels are significantly different between diagnosis and following the surgical tumor removal (between time points I and II) except miR-19a that appears to be more stable at time point II and decreases following the adjuvant therapy (time point III). Furthermore, the oncomiR’s serum level for healthy female is significantly lower than the oncomiR’s serum level at time point III of EBC patients.

### High-risk EBC patients express increased oncomiRs compared to low-risk EBC patients

We asked whether high-risk patients have different oncomiR serum levels compared to low-risk EBC patients, we first dichotomized EBC patients according to risk parameters (see Methods). Indeed, high-risk EBC patients possessed higher serum levels of oncomiRs’ (miR-155, miR-19a, miR-181b, and miR-24) than female-healthy controls (*p*-values 4.10e-06, 0.00256, 0.0059, and 0.00311) while the low-risk patients tend to have smaller oncomiRs’ values than the high-risk ones (*p*-values 0.026, 0.02567, 0.0250, and 0.00990). Therefore, it is strongly suggested that there might exist oncomiR level that distinguishes EBC patients according to risk for diagnostic purpose. Notably, the clinical data involving risk stratifications are in significant agreement with levels of all four oncomiRs (see Table 1A-D and Figure 1).The next question was how the levels of oncomiRs respond to therapy according to risk stratification. The serum level for each miR in case of high-risk patients (Figure 1, upper A panel) shows a decreasing trend through the therapy. The only exception is serum levels of miR-24, which was consistently low before and after therapy (between time points II and III). On the other hand, the oncomiRs of low-risk patients (Figure 1, bottom B panel) drop after the surgical resection and, consequently, remain almost constant during the therapy with approximately the same level as in case of controls.

To conclude, the levels of oncomiRs of high-risk patients drop less rapidly than low-risk patients. In addition, the high-risk patients do not achieve normal oncomiR levels as the low-risk patients do.

## Discussion

We herein present evidence that oncomiR levels are significantly different at initial diagnosis of EBC compared to time points following specific treatments (after surgery and after adjuvant therapy). Our results suggest that surgery had tremendous impact on three serum oncomiR levels while adjuvant therapy significantly decreased all of the four oncomiRs studied. This suggests that there exist relationship between EBC elimination throughout the therapy and serum oncomiR levels.

OncomiRs are often deregulated in solid cancers and leukemias and function in the gene-reprogramming of the cell to establish the phenotypic outcome. As such, their levels are accordingly detectable in patient sera at diagnosis and are thus strongly considered for diagnostic screening in cancer patients and their relatives [2]. Our other data confirm that BC tumor tissues indeed produced the studied oncomiRs and that their elevated levels in the sera reflect this (Additional file 2: Results 2). In addition, detection of oncomiRs can be useful for relapse detection however much longer follow up is needed. In our patient cohort, we observed BC relapse in three patients and this resulted in changes in oncomiR levels (Additional file 2: Results 3). However, as our cohort consisted of a low number of relapsed patients it did not allow more thorough analysis of oncomiRs. MiR-155 is one of the most studied miRs in BC patients and its levels were found consistently increased by others in the serum samples [15]. However, miR-155 production by a tumor is highly responsive to surgical therapy and therefore its levels decline very rapidly (similarly to our data). Contrary to this, miR-19a appears to be more stable and detectable following the surgical removal (as compared to miR-155, miR-181b, or miR-24). Finally, the levels of all studied oncomiRs decreased following the adjuvant therapy (time point III).

Our study demonstrated that the levels of oncomiRs for the high-risk patients’ drop less rapidly than in case of the low-risk patients. In addition, high-risk patients, unlike low-risk, do not achieve normal oncomiR levels following the adjuvant therapy (time point III). For example, miR-17-92 cluster appears to be very important factor in aggressive triple-negative BC [16]. Similarly, additional microRNA miR-181b was associated with more aggressive BC characterized by grading, Ki-67, and triple negativity (its pathogenic effect was linked to downregulation of ATM and influence on DNA damage response [17]). miR-24 has been associated with BC pathogenesis [19], but has not been so far suggested to be involved in BC aggressiveness. We also consider and cannot fully exclude the possibility that oncomiR levels drop less rapidly in high-risk patients due to post-surgical presence of tumor cells that are not however detectable by clinically-utilized techniques. However, both careful surgical removal of surrounding non-tumorous tissue as well as very low number of relapsed EBC patients suggests that the elevation reflects rather increased stability and possibly also slower turnover of the oncomiRs in the patient sera.

Longitudinal multivariate analysis of oncomiR expression in the EBC patient cohort strongly supports the clinical relevance of the four oncogenic microRNAs. In part it helps to associate observed deregulations in the serum samples with aggressive clinical parameters such as HER2, Ki-67, and grade III. This is consistent with previous analyses that were based on expression-data within tumor tissue [17] and some of the data with regard to miR-155 in sera were also consistent with others [20]. Our work thus provides important link in connecting biological function of miRs within the tumor with their levels in the sera.

## Conclusions

Our data provide evidence that the expression of four BC pathogenesis-related oncomiRs (miR-155, miR-19a, miR-181b, and miR-24) in sera are increased at diagnosis of EBC patients. These oncomiR levels decrease following combined therapy. Importantly, high-risk EBC patients show notably delayed and less-pronounced decrease of oncomiR expression following surgical tumor removal. Elevated expression of the oncomiRs in sera was also observed in primary tumor tissues. Upon relapse, serum levels of some oncomiRs are increased indicating their potential for the EBC patient monitoring. Multivariate analysis of the oncomiR expression in the EBC patient cohort suggested that adverse clinical characteristics associate with elevated oncomiRs in the EBC sera. OncomiR-profiling in sera is very promising tool to be incorporated in further studies and clinical practice as well.

## Abbreviations

EBC: Early breast cancer; miRs: microRNAs; oncomiRs: Oncogenic miRs; SOCS1: Suppressor of cytokine signaling-1; STAT3: Signal transducer and activator of transcription-3; PTEN: Phosphatase and tensin homolog; ATM: Ataxia-telangiectasia mutated; PARP1: Poly [ADP-ribose] polymerase 1; TGFβ: Tumor growth factor; HER2: Human epidermal growth factor receptor 2; ER: Estrogen receptor; PgR: Progesterone receptor; PCR: Polymerase chain reaction; SEM: Standard error of mean.

## Competing interests

The authors declare that they have no competing interests.

## Authors’ contributions

MS: patient sample collection, design of the study and writing the manuscript; PB carried out the molecular studies, participated in the experimental procedure development and drafted the manuscript; MP: statistical analysis, mathematical model, writing; ND: patient data analysis; JB participated in patient sample collection; PBu participated in sample processing; VP modified methods of miR isolation and detection and suggested let-7a as reference miR; TS: experimental design, data analysis and coordination and writing of the manuscript. All authors read and approved the final manuscript.

## Pre-publication history

The pre-publication history for this paper can be accessed here:

http://www.biomedcentral.com/1471-2407/14/448/prepub

## Supplementary Material

Additional file 1 **Patient prognostic and clinical markers, therapy, and survival data.** List of 63 patients with ID codes, clinical diagnosis and histology data. Prognostic and clinical markers, chosen therapy, survival data, and three cases of relapse are also indicated. Click here for file

Additional file 2 **Supplementary information for: Oncogenic MicroRNAs: miR-155, miR-19a, miR-181b, and miR-24 Enable Monitoring of Early Breast Cancer in Serum. Results 1** documenting that majority of EBC patients overexpresses 4 oncomiRs in sera at diagnosis of BC. **Results 2** show that BC tumor tissues produce oncomiRs and that the patients (providing these tumor samples) display elevated levels of these oncomiRs in the sera. **Results 3** show serum levels of oncomiRs upon clinical relapse. Click here for file
