# Supplementary material for: Oncogenic MicroRNAs: miR-155, miR-19a, miR-181b, and miR-24 enable monitoring of early breast cancer in serum
Source: BMC Cancer. 2014 Jun 18;14:448. doi: 10.1186/1471-2407-14-448 (PMC4075993; doi:10.1186/1471-2407-14-448)
Supplement: Additional file 2 — Supplementary information for: Oncogenic MicroRNAs: miR-155, miR-19a, miR-181b, and miR-24 Enable Monitoring of Early Breast Cancer in Serum. Results 1 documenting that majority of EBC patients overexpresses 4 oncomiRs in sera at diagnosis of BC. Results 2 show that BC tumor tissues produce oncomiRs and that the patients (providing these tumor samples) display elevated levels of these oncomiRs in the sera. Results 3 show serum levels of oncomiRs upon clinical relapse. [file 1471-2407-14-448-S2.pdf]

Supplementary information for:

Oncogenic MicroRNAs: miR-155, miR-19a, miR-181b, and miR-24 Enable Monitoring of Early Breast Cancer in Serum.

Marek Sochor[1*], Petra Basova[2*], Michal Pesta[3], Nina Dusilkova[2], Jiri Bartos[1], Pavel Burda[2], Vit Pospisil[2], and Tomas Stopka[2]

* These authors contributed equally to this work

Supplementary Results for SF1

Majority of EBC patients overexpresses 4 oncogenic miRs in sera at diagnosis of BC.

Expression profiles of the four serum oncomirs in EBC patients (N=63) were determined throughout the therapy response. We observed that individual oncogenic miRs exceed arbitrary 1.8 fold cut off (correspondingly to median fold-increase of miR-155 in BC tumor reported by [1]), expressed at diagnosis (Pre-Op, sample I) at the following frequencies; miR-155 in 43%, miR-19a in 38%, miR-181b in 33%, and miR-24 in 32% (SF1). Notably, the oncomiRs are often co-overexpressed in some but not all patients. Following surgery (sample II) and chemo-*or*-radiotherapy (sample III) the number of patients that had elevated levels of serum oncomiRs decreased (SF1, in boxes). For example for miR-155: 27 patients at diagnosis had increased miR-155 expression with 15 patients within the sample II cohort and only 7 patients in sample III. Controls of gene expression were healthy women that donated sera and additional microRNAs that are not oncogenic (let-7a). As most patients at sample III displayed *slightly increased-to-normal* oncomiR levels (as compared to healthy controls) and clinicians detected no tumor we concluded that oncomiRs-producing tumor cells were significantly depleted. Importantly, three relapsed patients (sample IV) displayed upregulated oncomiRs (SF1) indicating indeed that oncomiRs expression in the sera was correlated with re-overgrowth of the tumor.

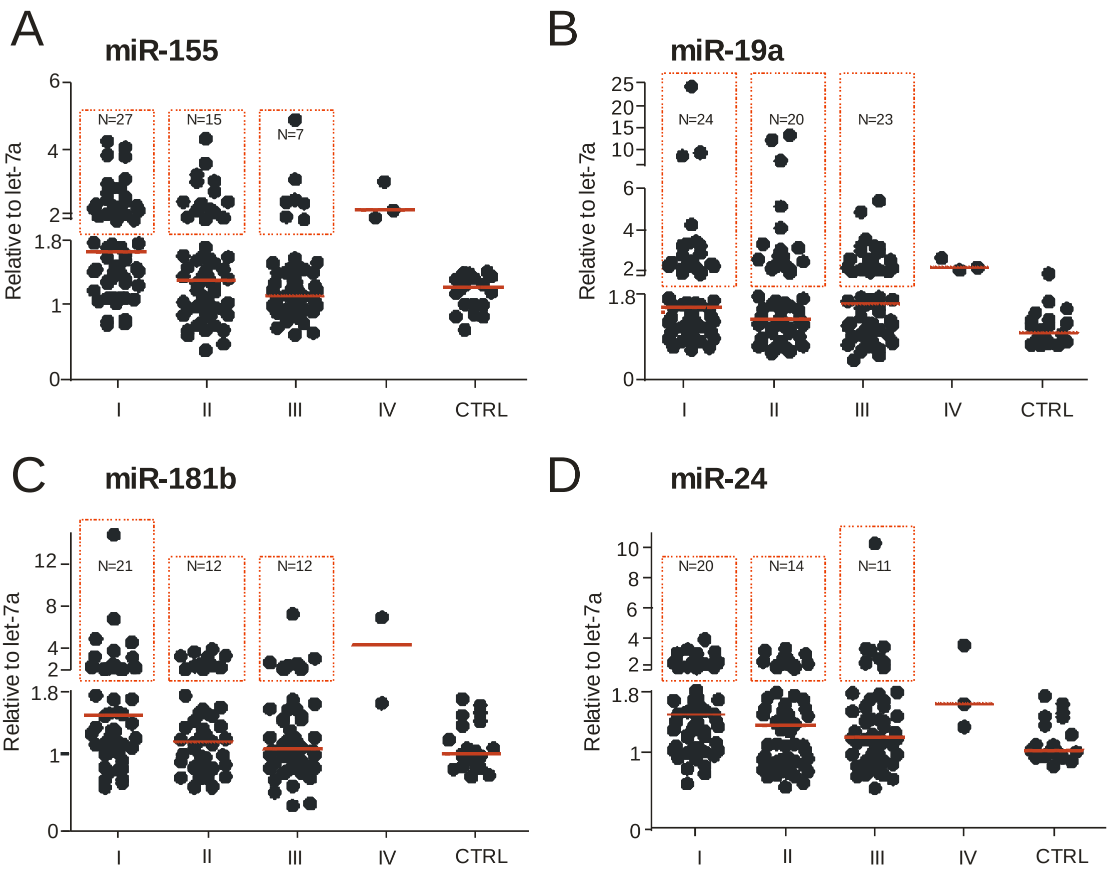

SF1 legend. Serum levels of oncomiRs in BC patients (N=63) and healthy female controls (N=21). 200 μL of sera was used to extract total RNA that was used for specific reverse transcription and TaqMan PCR amplification of miR-155 (A), miR-19a (B), miR-181b (C), and miR-24 (D) and also let-7a that served as normalization control. BC patients were analyzed at diagnosis (I), after surgical resection (II), and chemotherapy (III). Three patients were analyzed at relapse. Y-axis represents abundance of oncomiRs relative to non-oncogenic miR

(let-7a) in BC sera. Average values of healthy controls were set to 1. Numbers and % of patients in which abundance of miR>1.8 are shown in boxes.

Reference:

1. Iorio MV, Ferracin M, Liu CG, Veronese A, Spizzo R, Sabbioni S, Magri E, Pedriali M, Fabbri M, Campiglio M *et al*: **MicroRNA gene expression deregulation in human breast cancer**. *Cancer research* 2005, **65**(16):7065-7070.

Supplementary Results for SF2

BC tumor tissues produce oncomiRs and elevated levels in the sera reflect this.

To determine whether oncomiRs are indeed derived from EBC tumor tissue we studied closely 10 BC patients that provided a tumor sample during its surgical removal and also a sample of tumor-less breast tissue surrounding the tumor. Analysis of oncomiR expression in the BC tissue relative to the surrounding tissue identified 8/10 EBC patients showing oncomiR elevation (SF2A) comparably to serum levels (SF2B). Interestingly, high-risk BC patients express higher oncomiR levels (indicated by black circles, SF2A). Again, similarly to BC sera, BC tumors overexpressed one, two, or more oncomiRs indicating on heterogeneity of BC tumors in terms of oncomiR production. Based on expression data in the tumor we analyzed miR-155 in the sera of the three high-risk patients and observed that it was increased (compared to healthy control sera) at the time of diagnosis (I) and also, suggestive of more aggressive disease, within the two consecutive samples (II and III).

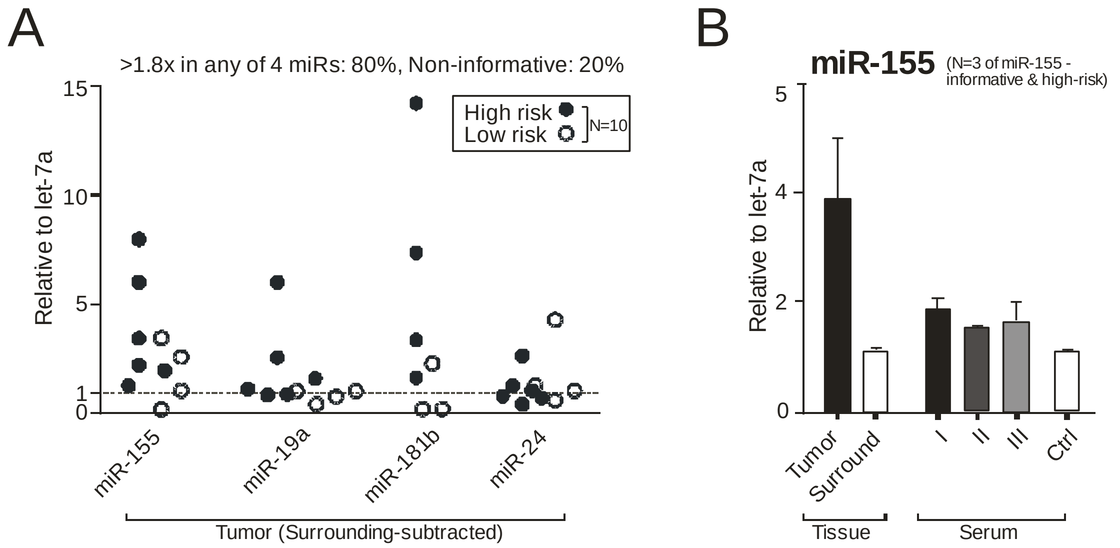

SF2 legend. Levels of oncomiRs in BC tumor tissue are increased compared to surrounding tissue. Tumor tissue and surrounding tissue not-affected by BC (N=10, left panel A) was removed by surgeon and lyzed to isolate total RNA that was used for specific reverse transcription and TaqMan PCR amplification of miR-155, miR-19a, miR-181b, miR-24, and also let-7a that served as normalization control. Risk parameters are indicated (high-risk: black circles, low-risk: empty circles). Informative high-risk BC patients (N=3, right panel B) also provided sera at the time of diagnosis (I), after surgical resection (II), and chemotherapy (III). Y-axis represents abundance of oncomiRs relative to non-oncogenic miR (let-7a) after subtracting values of surrounding tissue. Average values of controls were set to 1.

Supplementary Results for SF3

Serum levels of oncomiRs signal relapsed BC.

We next asked how the levels on oncomiRs in sera respond to a patient relapse. Due to lower frequency of EBCs relapse we analyzed three relapsed patients (SF3). First patient relapsed at the site of original tumor and the levels of all four oncomiRs in the sample IV were increased (SF3A) and this was signaled also in the sample II suggesting the possibility that the tumor was not completely surgically removed. Second patient presented with contralateral relapse (SF3B). Again, this patient shows significant oncomiR serum elevation at sample II and a decrease upon systemic therapy (sample III). Interestingly, miR-181b and miR-19a were not fully normalized at sample III. Relapse is in this patient was associated with spiked increased in the serum miR-181b level. Third patient relapsed by systemic metastasis (SF3C). This was again characterized by increased oncomiRs in sample II and their continuous elevation upon clinical relapse.

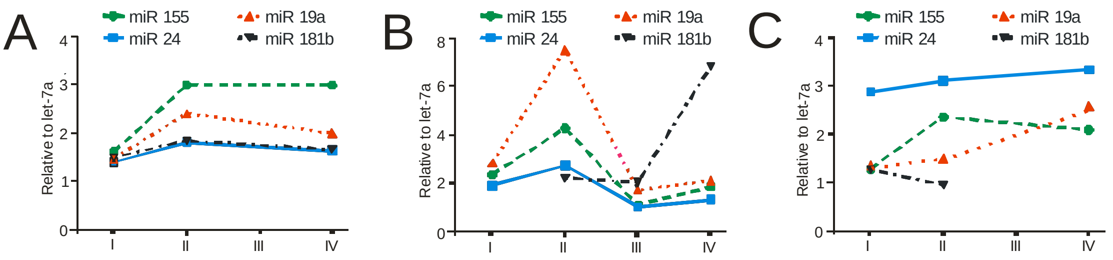

SF3 legend. Serum levels of oncomiRs signal relapsed BC. Three relapsed BC patients were analyzed at diagnosis (I), after surgical resection (II), after chemotherapy (III), and upon relapse (IV). A: local relapse at the original site of tumor, B: contralateral relapsed tumor, C: systemic metastasis of BC. MiR-155 (circles, dashed line), miR-19a (triangles, dot line), miR-181b (flipped triangle, dots&dashed line), and miR-24 (rectangle, cont. line) were detected in BC sera (see M&M). Y-axis represents abundance of oncomiRs relative to non-oncogenic miR (let-7a) in BC sera and relative to healthy controls.
